# Hayes Yard virus: a novel ephemerovirus isolated from a bull with severe clinical signs of bovine ephemeral fever is most closely related to Puchong virus

**DOI:** 10.1186/s13567-020-00781-1

**Published:** 2020-04-29

**Authors:** Kim R. Blasdell, Steven S. Davis, Rhonda Voysey, Dieter M. Bulach, Deborah Middleton, Sinead Williams, Margaret B. Harmsen, Richard P. Weir, Sandra Crameri, Susan J. Walsh, Grantley R. Peck, Robert B. Tesh, David B. Boyle, Lorna F. Melville, Peter J. Walker

**Affiliations:** 1 CSIRO Health and Biosecurity, Australian Animal Health Laboratory, 5 Portarlington Road, Geelong, VIC 3220 Australia; 2 Berrimah Veterinary Laboratories, Department of Primary Industry and Fisheries, Darwin, NT Australia; 3 Timor-Leste Office, Menzies School of Health Research, Dili, Timor-Leste; 4 Melbourne Bioinformatics, The University of Melbourne, Carlton, VIC 3053 Australia; 5 Center for Biodefense and Emerging Infectious Diseases, Department of Pathology, University of Texas Medical Branch, Galveston, TX USA; 6 School of Chemistry and Biomolecular Sciences, The University of Queensland, St Lucia, QLD 4072 Australia

## Abstract

Bovine ephemeral fever is a vector-borne disease of ruminants that occurs in tropical and sub-tropical regions of Africa, Asia and Australia. The disease is caused by a rhabdovirus, bovine ephemeral fever virus (BEFV), which occurs as a single serotype globally. Although several other closely related ephemeroviruses have been isolated from cattle and/or arthropods, only kotonkan virus from Nigeria and (tentatively) Mavingoni virus from Mayotte Island in the Indian Ocean have been previously associated with febrile disease. Here, we report the isolation of a novel virus (Hayes Yard virus; HYV) from blood collected in February 2000 from a bull (*Bos indicus*) in the Northern Territory of Australia. The animal was suffering from a severe ephemeral fever-like illness with neurological involvement, including recumbency and paralysis, and was euthanised. Histological examination of spinal cord and lung tissue identified extensive haemorrhage in the dura mata with moderate perineuronal oedema and extensive emphysema. HYV displayed cone-shaped morphology, typical of rhabdoviruses, and was found to be most closely related antigenically to Puchong virus (PUCV), isolated in 1965 from mosquitoes in Malaysia. Analysis of complete genome sequences of HYV (15 025 nt) and PUCV (14 932 nt) indicated that each has a complex organisation (3′ N-P-M-G-G_NS_-α1-α2-β-γ-L 5′) and expression strategy, similar to that of BEFV. Based on an alignment of complete L protein sequences, HYV and PUCV cluster with other rhabdoviruses in the genus *Ephemerovirus* and appear to represent two new species. Neutralising antibody to HYV was also detected in a retrospective survey of cattle sera collected in the Northern Territory.

## Introduction

The genus *Ephemerovirus*, family *Rhabdoviridae*, comprises negative-sense, single-stranded RNA viruses that infect primarily ruminants and are transmitted by haematophagous insects [1, 2]. Bovine ephemeral fever virus (BEFV) is the type member of the genus (species *Bovine fever ephemerovirus*) [1]. BEFV is an important pathogen of cattle and water buffalo, causing a disease of rapid onset characterised by a bi‐phasic fever, salivation, ocular and nasal discharge, recumbency, muscle stiffness, lameness and anorexia. [3]. It occurs seasonally in many tropical and sub-tropical regions of Africa, Asia (including the Middle-East) and Australia, but remains exotic to the Americas, the Pacific Islands and almost all of Europe [3]. High infection and morbidity rates typically occur in cattle during BEFV epizootics. Mortality rates are usually low (>1%), although there have been several reports of much higher mortality from some regions in recent years [4–6]. The primary economic impacts of the bovine ephemeral fever (BEF) disease are on milk production in dairy herds, meat quality in beef herds and farm traction power provided by water buffalo [7–9]. BEFV has been isolated on multiple occasions from diseased cattle, and both mosquitoes and biting midges (*Culicoides* spp.); the principal insect vectors appear to vary in different regions of the world [3].

Eight other ephemeroviruses, each distinguishable in virus neutralisation tests, have been isolated in Africa or Australia [2, 10]. However, only kotonkan virus (KOTV; species *Kotonkan ephemerovirus*), isolated from biting midges in Nigeria in 1967, has been associated with clinical disease [11]. At that time, a serological survey indicated a high prevalence of KOTV-neutralising antibodies in cattle, including five imported Friesian heifers that had recently shown clinical signs of bovine ephemeral fever [11]. Mild clinical signs were also observed in one of two native white Fulani calves infected experimentally with mouse-brain-passaged KOTV [12]. To our knowledge, there have been no other reports of KOTV-related disease in Africa or elsewhere. However recently, Mavingoni virus (MVGV), which potentially could be assigned to a new species within the genus *Ephemerovirus*, was detected in a bovid with BEF-like symptoms from Mayotte Island in the Indian Ocean [13].

Of the other known ephemeroviruses, Berrimah virus (BRMV; species *Berrimah ephemerovirus*), Kimberley virus (KIMV; species *Kimberley ephemerovirus*), Adelaide River virus (ARV; species *Adelaide River ephemerovirus*) and Koolpinyah virus (KOOLV; species *Koolpinyah ephemerovirus*), have each been isolated from healthy sentinel cattle in Australia but have never been associated with clinical disease [14–17]. Serological surveys have indicated that KIMV and ARV are widespread in Australia, and also occur in Indonesia, Papua New Guinea and China, where they appear to cause only sub-clinical infections in cattle [14, 18–20]. Obodhiang virus (OBOV; species *Obodhiang ephemerovirus*), Yata virus (YATV; species *Yata ephemerovirus*) and Malakal virus (MALV; later recognised as KIMV) were each isolated in Africa from mosquitoes (*Mansonia uniformis*) during the 1960s but their host range and distribution remain unknown [21–24]. Similarly, Puchong virus (PUCV; not formally unclassified) was isolated from mosquitoes (*Mansonia uniformis*) in Malaysia in 1965 [21]. Although PUCV has been identified antigenically as a member of the BEFV serogroup, little is known of its vertebrate hosts or distribution [10]. Despite a lack of evidence of disease association of these viruses, there have been anecdotal reports from investigating veterinarians that cases of bovine ephemeral fever do occur (in Australia at least), in the absence of seroconversion to BEFV neutralising antibodies [2].

Here, we report retrospectively the isolation and characterisation of a novel ephemerovirus (Hayes Yard virus; HYV) from a bull (*Bos indicus*) in Australia which, in February 2000, had displayed severe clinical signs resembling bovine ephemeral fever. We show that HYV is most closely related to PUCV from Malaysia and provide evidence that HYV-neutralising antibodies occur in Australian cattle.

## Materials and methods

### Viruses and cells

Passage histories of ARV, BEFV, BRMV, KIMV, KOTV, MALV and OBOV used for the virus neutralisation tests have been described previously [22, 23]. HYV and PUCV were grown at 37 °C in baby hamster kidney (BHK-BSR) cells in Basal Medium Eagle (BME) supplemented with 10 mM HEPES, 2 mM l-glutamine, 137 μM streptomycin, 80 U/mL penicillin, and either 5% (growth medium) or 2.5% (maintenance medium) foetal calf serum. Prior to sequencing, HYV was passaged once in *Aedes albopictus* mosquito (C6–36) cells and four times in BHK-BSR cells. PUCV was passaged six times in suckling mice, once in C6–36 cells and three times in BHK-BSR cells.

### Antisera

ARV rabbit antiserum, BEFV bovine immune serum, BRMV, KIMV, KOTV, MALV and OBOV immune mouse ascetic fluids (IMAFs) and negative control bovine serum have all been described previously [22, 23]. PUCV IMAF was produced as described previously [25]. Experimentally produced antiserum was not available for HYV.

### Transmission electron microscopy (TEM)

BHK-BSR cells inoculated with HYV were pelleted in a bench top centrifuge at 840 ×* g* for 1 min and the supernatant media removed for negative contrast TEM. The pellets were processed, thin sectioned and stained as described previously [26] except using 0.1 M Sorenson’s phosphate buffer for dilution of osmium tetroxide. The reserved supernatant was prepared for negative contrast electron microscopy (EM) [26]. Grids were examined using a Philips CM120 or a JEOL JEM1400 transmission electron microscope at 120 kV.

### Preparation of viruses for next generation sequencing (NGS)

Supernatant was collected from HYV-infected BHK-BSR cells when cytopathic effect was advanced, clarified by centrifugation at 3200 × *g* for 5 min and passed through a 0.45 µM filter. The clarified supernatant was added (3:1 by volume) to a solution of 30% polyethylene glycol and 23% NaCl (diluted in TEN buffer), incubated overnight and then centrifuged at 3200 × *g* for 25 min at 4 °C. The supernatant was removed and the pellet resuspended in 50 µL TEN buffer prior to treatment with 2 U of Turbo™ DNase (Thermo Fisher Scientific, Waltham, USA) at 37 °C for 30 min followed by inactivation at 75 °C for 10 min, as per the manufacturer’s instructions. Total RNA was then extracted using Trizol reagent (Thermo Fisher Scientific) followed by first strand cDNA synthesis with Superscript III (Thermo Fisher Scientific) and second strand synthesis with Klenow large fragment (NEB, Ipswich, USA), both with K-8N primers as described previously [27]. A sequence-independent single primer amplification (SISPA) PCR was performed in quadruplicate on the double-stranded cDNA using LongAmp DNA polymerase (NEB) and primer 8 essentially as described previously [27], but with the following cycle: 94 °C for 2 min; 94 °C for 30 s, 54 °C for 30 s, 65 °C for 1 min, all for 20 cycles; 65 °C 10 min; and standing at 4 °C. PCR products were combined and run on a 1.5% agarose gel. The smear observed in the 200–800 bp region was excised and the DNA purified using the QIAquick gel extraction kit (Qiagen, Hilden, Germany). Purified SISPA products were quantified on a Qubit™ (Thermo Fisher Scientific) using the Qubit™ dsDNA HS assay kit (Thermo Fisher Scientific), and then prepared for sequencing using the Nextera^®^ XT DNA library preparation kit (Illumina, San Diego, USA) as per the manufacturer’s instructions.

For PUCV, total RNA extraction from infected cells and a modified PCR-select cDNA subtraction for enrichment of viral sequences were carried out as described previously [28, 29]. Wongabel virus (WONV; species *Wongabel hapavirus*) was used as the driver for the PCR-select method and three restriction enzymes (*Alu* I, *Hae* III, *Rsa* I) were used for library construction.

### Next Generation Sequencing (NGS) and analysis

For HYV, sequencing was performed using a MiSeq Reagent kit v2 (300 cycles) (Illumina) on an Illumina MiSeq platform. The CLC Genomics Workbench software package version 9.5.2 was used for quality assessment, trimming and *de novo* assembly of contigs for HYV. For PUCV, the Illumina GAIIx platform at Micromon (Monash University, Clayton, Australia) using the manufacturer’s protocol was used to perform high-throughput DNA sequencing. Subsequent analyses were conducted as described previously [22].

### Rapid amplification of cDNA ends (RACE)

The RACE protocol has been described previously [22, 30]; primers used are shown in Additional file 1. Sanger sequencing of RACE products was performed using the BigDye Terminator v. 3.1 (Applied Biosystems, Foster City, USA) and the ABI PRISM 3130xl Genetic Analyser.

### Phylogenetic analysis

An alignment of 125 complete rhabdovirus L protein sequences was created using MUCSLE in MEGA version 7.0 [31] and ambiguously aligned regions were removed using Gblocks [32]. The resulting alignment comprising 563 amino acids was used to infer phylogenetic relationships in MEGA 7.0 using the Maximum Likelihood method and the WAG + Frequency model of amino acid substitution [33]. The phylogenetic robustness of each node was determined using 1000 bootstrap replicates. Trees were annotated using Figtree version 1.4.2.

### Transcription profiling

Transcription profiling was performed using PCR as described previously [22]. The PCR primers and annealing temperatures used are shown in Additional file 1.

### Radiolabelling, SDS-PAGE and immunoblotting

Radiolabelling, immunoblotting and SDS-PAGE were performed essentially as described previously [22, 28]. For immunoblotting, both the primary (PUCV IMAF) and the secondary (HRP-conjugated sheep anti-mouse IgG; Silenus, Hong Kong, China) antibodies were used at 1:10 000 dilution.

### Virus cross-neutralisation tests

Virus neutralisation tests were performed as described previously [22, 34]. Cross-neutralisation antibody titres were determined as the lowest dilution of antibody at which neutralisation was observed and were calculated using the method of Reed and Muench [35].

### Serum neutralisation assays: field sera

Assays were conducted using a modified version of the serum neutralisation test described previously [36]. Briefly, 50 µL of serially diluted serum, in quadruplicate wells, was incubated with 50 µL of medium containing 100 TCID_50_ of virus for 1 h at 37 °C in 96 well plates. Then 100 µL of medium containing BHK-BSR cells at 2 × 10^5^ cells/mL was added and the plates were read after incubation for a further 5 days at 37 °C. Serum neutralisation titres were calculated using the method of Reed and Muench [35]. Titres below 1:10 were deemed negative.

### PUCV G protein ELISA

An indirect ELISA targeting the PUCV glycoprotein (G protein) was developed by modifying a previously published method [37] prior to the identification of HYV. The ELISA targets the linear G1 neutralising epitope which is known to vary among ephemeroviruses. However, due to the cross-reactivity observed between PUCV and HYV during virus cross-neutralisation tests, it was expected that this assay would be able to detect both PUCV and HYV antibodies. The region of the PUCV G protein gene corresponding to the BEFV G1 epitope was amplified using *Pfu* DNA polymerase (Promega, Madison, USA) and PCR primers PUCV_TOPO_F (**CACC**TACACAAGAGCATTATGTGAG) and PUCV_TOPO_R (**CTA**CCAACCTACAATCCCGGAAAC), with the forward primer incorporating the TOPO cut site and the reverse primer a stop codon, both shown in bold. The amplicon was sequenced prior to cloning and protein expression using the Champion™ pET151 Directional TOPO^®^ expression kit (Thermo Fisher Scientific) according to the manufacturer’s protocol. Plasmids were purified using PureYield™ plasmid miniprep system (Promega) according to the manufacturer’s protocol. Protein expression was confirmed by Coomassie Brilliant Blue staining and immunoblotting using mouse anti-V5 antibody (Thermo Fisher Scientific) at 1:5000 and secondary antibody (HRP-conjugated sheep anti-mouse IgG; Sigma, St. Louis, USA) at 1:5000. Recombinant fusion proteins were purified using BugBuster^®^ Mastermix (Merc Millipore, Burlington, USA) and ProPur™ IMAC midi spin columns (Nunc, Roskilde, Denmark), followed by desalting and concentration in Vivaspin™ 20 protein concentrator spin columns (GE Healthcare, Chicago, USA). Checkerboard titrations were used to select the concentration of G1 antigen and the dilutions of the positive control and secondary antibody for use in ELISA. G1 antigen was coated onto ELISA plates at 250 ng/well, PUCV IMAF was used as the positive control at dilutions of 1:100 and 1:200, and HRP-conjugated protein A/G (Thermo Fisher Scientific) was used as the secondary antibody at a dilution of 1:20 000 in blocking buffer (PBS with 5% skimmed milk powder and 0.05% Tween). Optical density was determined using a 450 nm absorbance filter. Bovine field sera were tested at dilutions of 1:20 and 1:40. Based on the performance of this assay with known negative samples, at both dilutions, positive to negative reading ratios of >20 were considered positive and between >10 and 19 as indeterminate. Cross-reactions were not observed using antisera generated against other ephemeroviruses, including ARV, BEFV, BRMV, KIMV, KOTV, MALV, OBOV and YATV.

### Inoculation of experimental animals with PUCV

Experimental infection of four calves (3–4 months of age) was conducted under protocol AAEC #1569, which was endorsed by the CSIRO AAHL Animal Ethics Committee. Baseline blood samples were collected and data loggers fitted to all animals prior to inoculation and animals were allowed to acclimatise for 7 days. Each animal was inoculated intravenously with 2.2 mL of PUCV at a dose of 10^6.43^ TCID_50_/mL. Animals were monitored and temperature readings collected for 3 weeks after inoculation. Blood samples were collected daily between 1 and 7 days post-inoculation (dpi), and on the day of termination of the experiment. RNA was extracted from blood samples using the QIAmp RNA blood mini kit (Qiagen) and cDNA synthesised using Superscript III reverse transcriptase (Thermo Fisher Scientific). A previously described generic ephemerovirus RT-PCR assay [38] was used to detect PUCV RNA. Detection of anti-PUCV antibodies was performed using the PUCV G protein ELISA (described above).

## Results

### Clinical signs, histopathology and virus isolation

On 25 February 2000, diagnostic samples were received from a moribund 2.5 year-old bull (*Bos indicus*), from Beatrice Hill Farm, approximately 50 km south-east of Darwin, Australia. The bull had been suffering for 3 days from an unknown illness with neurological involvement, including recumbency and paralysis, and was euthanized due to the severity of the illness. Histological examination of spinal cord and lung tissue identified extensive haemorrhage in the dura mata with moderate perineuronal oedema and extensive emphysema with mixed inflammatory cells in alveolar septae. There were no histological lesions indicative of babesia infection or transmissible spongiform encephalopathy (TSE). Blood samples tested negative by polymerase chain reaction (PCR) for BEFV [39]. Passage of a sample of EDTA-blood in C6/36 cells resulted in the isolation of an unidentified virus (DPP4816) which was subsequently adapted to growth in BHK-BSR cells. Viral titres following passage in cell culture were consistently quite low (range: 10^3.38^–10^5.43^ TCID_50_/mL). Antibody titres in serum collected on the day of post-mortem were 1/160 for BEFV and 1/10 for DPP4816.

### Virus identification

DPP4816 was subsequently included in a retrospective analysis of unidentified virus isolates from the Northern Territory by using an RT-PCR targeting the L genes ephemerovirus and related rhabdoviruses [38]. Sequence analysis of PCR amplicons from 21 of the samples identified 12 as BEFV and 8 were identified as KIMV. One of the isolates (DPP4816, also referred to as V4816) was identified as a previously unknown ephemerovirus, designated Hayes Yard virus (HYV). Phylogenetic analysis of the translated partial L protein sequences (100 nt amplicon) suggested that the virus was most closely related to PUCV from Malaysia (Additional file 2).

### Virus morphology

**Figure 1:**
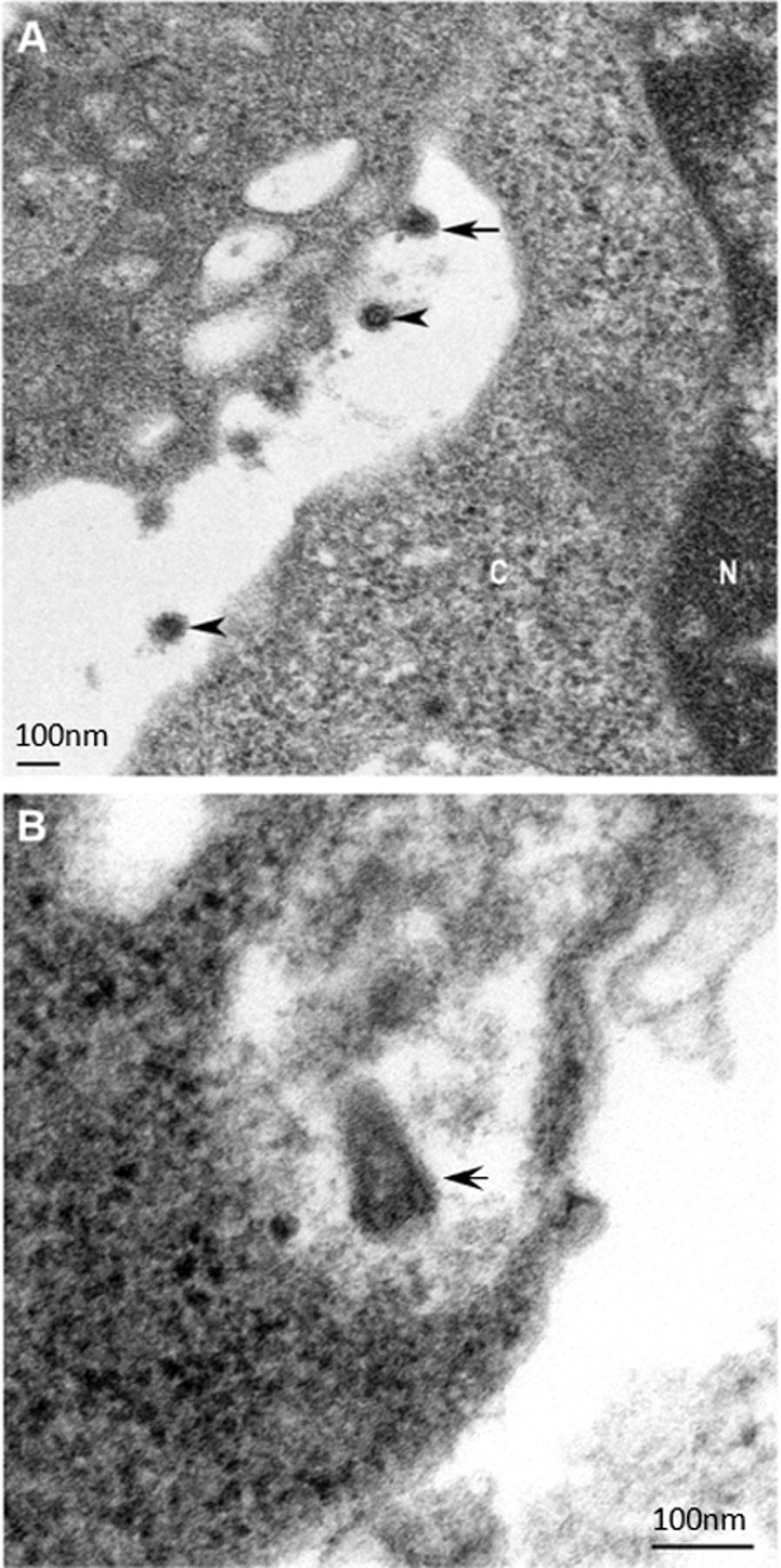
**Thin section transmission electron micrographs of BHK-BSR cells infected with Hayes Yard virus. A** Top arrow indicates a bullet-shaped virus budding from the plasma membrane. The bottom two arrows indicate transversely sectioned virus in the extracellular space. C: cytoplasm, N: nucleus. **B** The arrow indicates a cone- or bullet-shaped particle located at the surface of the cell. Scale bars (left bottom corner of **A** and right bottom corner of **B**) represent 100 nm.

Transmission electron microscopy of HYV-infected BHK-BSR cells revealed cone-shaped rhabdovirus-like particles, approximately 75 nm diameter at the base and approximately 150 nm in length, located at the cell surface (Figure 1A). Virions were also observed budding from the plasma membrane (Figure 1B).

### Nucleotide sequences of the HYV and PUCV genomes

**Figure 2:**
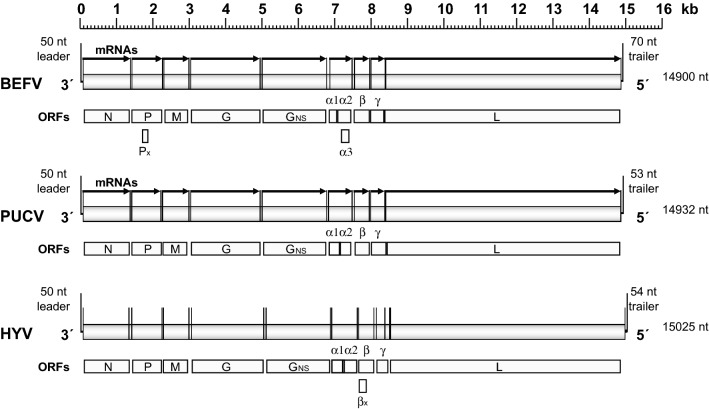
**Schematic illustration of the genome organisations of BEFV, PUCV and HYV.** The genomes are shown in 3′ to 5′ polarity with conserved transcription initiation and transcription termination/polyadenylation signals shown as vertical lines flanking open reading frames (ORFs). The locations of mRNAs identified experimentally are shown for BEFV and PUCV.

The complete genome sequences of the HYV (15 025 nt) and PUCV (14 932 nt) were determined by deep sequencing, with the terminal sequences resolved by using RACE. The sequences have been deposited in GenBank under Accession numbers MH507506 (HYV) and MH507505 (PUCV). The total number of reads mapping to PUCV and HYV were 1 791 000 and 3 959 831 respectively and the average coverage was ~8000 and ~32 000 respectively. For each virus, the genome organisation was found to be similar to that of BEFV, BRMV and KIMV, with the 5 cannonical rhabdovirus structural protein genes (*N*, *P*, *M*, *G* and *L*) and a long region between the *G* and *L* genes containing additional ORFs encoding a non-structural glycoprotein (G_NS_), a viroporin (α1) and three putative accessory proteins (α2, β and γ), currently of unknown function (Figure 2). Each additional ORF occurs within an independent transcriptional unit bounded by conserved transcription initiation (TI) and transcription termination/polyadenylation (TTP) sequences, except for ORF α2 which overlaps ORF α1 within the same transcriptional unit. As in several other ephemeroviruses, HYV and PUCV each feature a “termination upstream ribosome-binding site” (TURBS)-like sequence (UGGGA in mRNA polarity) in the α1/α2 ORF overlap region [40]. Unlike BEFV, neither the HYV or PUCV genomes contain alternative open reading frames within the P ORF (i.e., P′) or α2 ORF (i.e., α3), but there is a small alternative ORF (120 nt) that commences near the start of the HYV β gene that encodes a possible protein of 6.6 kDa (designated βx); it is not known if polypeptides encoded in any of these small ORFs are expressed during infection. We also note that the sequence in the region of the HYV G gene transcription termination sequence featured a stretch of up to 15 adenosine residues, for which the precise number could not be determined unambiguously.

Clustal W pairwise alignments of the whole genomes indicated that HYV and PUCV share 68.8% nucleotide sequence identity (p-distance estimated in MEGA 7.0) (Additional file 3). This level of identity is similar to BEFV and BRMV (67.0% identity) which are the most closely related ephemeroviruses currently assigned to different species. In contrast, KIMV and MALV, which are considered to be geographic variants of the same virus (and are assigned to the same species, *Kimberley ephemerovirus*) share 90.7% nucleotide sequence identity across the entire genomes (Additional file 3).

### Deduced amino acid sequences of HYV and PUCV proteins

Based on the deduced amino acid sequences, the predicted molecular weights of the unmodified structural proteins HYV and PUCV are 49.3 kDa and 49.0 kDa (N proteins), 31.4 kDa and 30.5 kDa (P proteins), 25.4 kDa and 25.4 kDa (M proteins), 75.1 kDa and 74.7 kDa (G proteins) and 243.6 and 246.9 kDa (L proteins), respectively. Comparison of amino acid sequence identities (p-distances) of the N, G and L proteins (Additional file 3) confirmed whole genome comparisons that indicated HYV and PUCV are more closely related to each other than to any other ephemerovirus (95.5% identity, 80.8% and 87.7% identity, respectively), except for KIMV and MALV (98.6%, 95.6% and 96.9%, respectively) which are assigned to the same ephemerovirus species.

A sequence alignment of the BEFV G protein with those of HYV and PUCV indicated that all cysteine residues in the ectodomain are conserved (Additional file 4). These include the twelve core cysteine residues which also occur in the G protein of vesicular stomatitis Indiana virus, where they form six disulphide bridges (C_I_–C_XII_, C_II_–C_IV_, C_III_–C_V_, C_VI_–C_VII_, C_VIII_–C_X_ and C_IX_–C_XI_) that assist in maintaining the secondary structure of the folded protein [2, 41], and six additional cysteine residues that are likely to form three additional disulphide bridges (a–f, b–c and d–e) in the ephemerovirus G proteins [2, 42]. Furthermore, alignment of the BEFV G protein and large non-structural glycoprotein (G_NS_) with those of HYV and PUCV indicated that ten of the 12 core cysteine residues and four of the six additional cysteine residues are conserved; only disulphide bridges (C_VI_–C_VII_) and (d–e) appear to be absent, indicating extensive similarities in secondary structure of the folded ephemerovirus G and G_NS_ proteins.

Other HYV and PUCV putative accessory proteins (α1, α2, β and γ proteins) are similar in size to and share extensive sequence homology with the cognate BEFV proteins (Additional file 5). Like BEFV α1, the HYV and PUCV α1 proteins (14.8 kDa and 14.5 kDa, respectively) have the structural characteristics of class 1a viroporins, with a central hydrophobic transmembrane domain, a highly basic C-terminal domain and clusters of large aromatic residues (W, F) in the N-terminal domain. The HYV and PUCV α2 proteins (15.4 kDa and 15.5 kDa, respectively) are neutral in charge (pI ~7.5); they share 73.9% amino acid sequence identity (p-distance) but much lower identity with BEFV α2 (24.3% and 22.6%, respectively). The HYV and PUCV β proteins (16.7 kDa and 16.6 kDa, respectively) are moderately basic (pI ~9.0); they share a high level of sequence identity (96.3%) and significantly lower identity with BEFV β (55.5% and 57.5%, respectively). The HYV and PUCV and γ proteins (13.2 kDa and 13.4 kDa, respectively) are also moderately basic (pI ~9.2); they share a high level of sequence identity (93.6%) and lower identity with BEFV γ (63.7% and 60.0%, respectively). The α2, β and γ proteins appear to be unique to ephemeroviruses but have no recognisable structural characteristics that would suggest their functions.

### PUCV gene expression profiles

**Figure 3:**
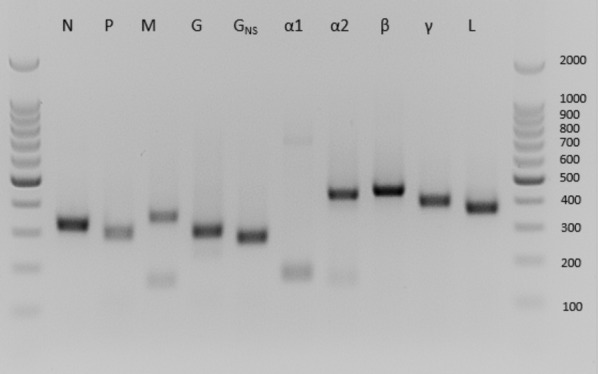
**PUCV viral transcription profiling.** Detection of PUCV transcripts in infected BHK-BSR cells by anchor PCR using an oligo(dT) primer and a sequence-specific primer in each ORF.

As HYV grew poorly in cell culture making downstream analysis unfeasible, expression analysis was conducted only for PUCV. Viral transcripts were identified in total RNA extracted from PUCV-infected BHK-BSR cells by anchor PCR utilising an oligo(dT) primer and sequence-specific primers targeting each ORF (Figure 3). Sequence analysis of the amplicons indicated that, except ORFs α1 and α2, each ORF is expressed as a monocistronic mRNA with transcription initiating and terminating at the immediately flanking TI and TTP sequences. In the case of the α1 ORF, a smaller product (<200 base pairs) was due to non-specific amplification and a larger product (750 base pairs) corresponded to an amplicon that terminated at the TTP sequence following the PUCV α2 ORF. The amplicon generated using the sequence-specific primer in the α2 ORF also terminated at this same TTP sequence. The data indicate that, as in several other ephemeroviruses, the α1 and α2 ORFs (which are not separated by TTP and TI sequences) are expressed from a bicistronic mRNA. Non-specific amplification is also likely the cause of the smaller fainter product observed for M.

**Figure 4:**
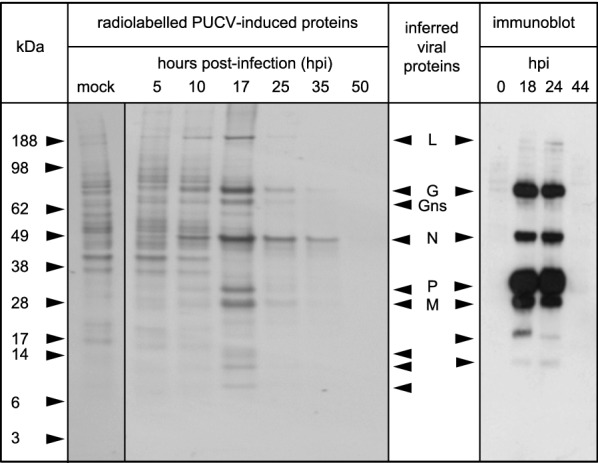
**PUCV protein expression profiling.** SDS-PAGE of proteins expressed in PUCV-infected BHK-BSR cells detected by pulse radiolabelling of proteins with L[^35^S]-methionine and L[^35^S]-cysteine for 1 h at various times post-infection (left panel); or by immunoblotting using PUCV-specific mouse immune ascites fluid at various times post-infection (right panel). Bands corresponding approximately in size to the estimated molecular masses of the major viral structural proteins (L, G, N, P and M) and the non-structural glycoprotein (G_NS_) are indicated. Other bands that appear to be induced or were detected by immunoblotting in infected cells but not mock-infected cells are also indicated with arrowheads.

To identify viral-induced proteins, BHK-BSR cells were inoculated with PUCV at a multiplicity of infection (MOI) of 3 TCID_50_/cell. PUCV-infected BHK-BSR cells and mock-infected control cells were pulsed for 1 h with L[^35^S]-methionine/cysteine at 5, 10, 17, 25, 35 and 50 h post-infection (hpi) and analysed by SDS-PAGE and autoradiography (Figure 4). A marked shutdown of host cell protein synthesis was observed in PUCV-infected cells from 17 hpi but, as infection progressed, several protein bands of increasing intensity were observed with relative migrations (i.e. molecular mass (Mr)) of approximately 197, 78, 68, 47, 33 and 29 kDa; these corresponded roughly to the predicted molecular weights of the PUCV L, G, G_NS_, N, P and M proteins. In addition, there were several PUCV-induced protein bands with Mr of approximately 15 kDa, 13 kDa and 9 kDa and these may correspond to several of the smaller proteins encoded in the α1, α2, β or γ ORFs. SDS-PAGE and immunoblotting with anti-PUCV IMAF of proteins expressed from PUCV-infected BHK-BSR cells (inoculated at MOI = 3) also identified protein bands induced at 18 and 24 hpi that appeared to correspond to the PUCV structural proteins L, G, N, P and M (Figure 4). Two smaller induced proteins did not appear to correspond in size to proteins detected in the pulse labelling experiment and may have been breakdown products of the viral structural proteins.

### Phylogenetic relationships

**Figure 5:**
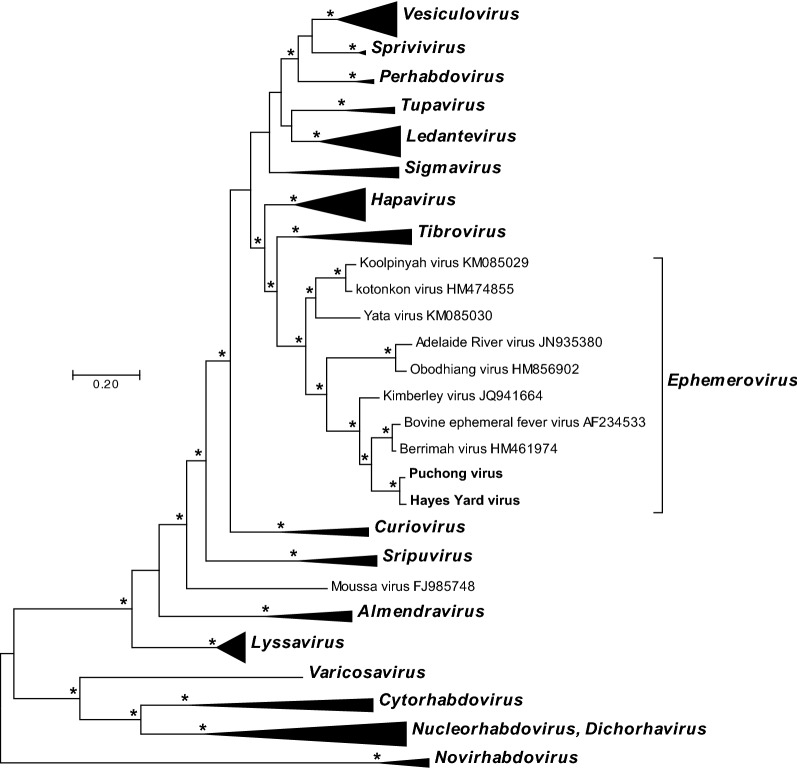
**Phylogenetic analysis of HYV and PUCV.** A phylogenetic tree inferred from a MUSCLE alignment of complete L protein sequences of 126 rhabdoviruses currently assigned to species, as well as HYV and PUCV. Phylogenetically informative sites were selected from the alignment using Gblocks resulting in 565 positions in the final dataset. The tree was inferred in MEGA version 7.0.18 by using the Maximum Likelihood method. The tree with the highest log likelihood (−59 565.1689) is shown. Initial tree(s) for the heuristic search were obtained automatically by applying Neighbor-Join and BioNJ algorithms to a matrix of pairwise distances estimated using a JTT model, and then selecting the topology with superior log likelihood value. The tree is drawn to scale, with branch lengths measured in the number of substitutions per site. Nodes with bootstrap support values (1000 iterations) >70% are indicated (*). (Note that viruses currently assigned to the genus *Nucleorhabdovirus* and the genus *Dichorhavirus* are shown as a single cluster as the genera do not represent monophyletic groups).

A Maximum Likelihood phylogenetic tree was inferred from a MUSCLE alignment of the complete L protein sequences of HYV, PUCV and rhabdoviruses representing all currently assigned genera and species in the *Rhabdoviridae* (Figure 5). HYV and PUCV fell together within the ephemerovirus clade, supported by strong bootstrap values. HYV and PUCV clustered most closely with BEFV and BRMV in a subclade that also included KIMV.

### Antigenic relationships

Antigenic relationships between PUCV and several other ephemeroviruses (BEFV, BRMV, KIMV, ARV, OBOV and KOTV) were determined by virus neutralisation tests (Table 1). As observed previously, low-level cross-neutralisation was observed between BEFV and BRMV and between ARV and OBOV. Very low-level cross-neutralisation of KIMV and OBOV (titre 1/20) was also detected using anti-PUCV IMAF (homologous titre 1/5120). Virus neutralisation tests were also conducted to determine the antigenic relationship between PUCV and HYV by using anti-PUCV IMAF, anti-BEFV and anti-KIMV bovine sera and three sera found to be positive by a PUCV G protein ELISA (Table 2). Anti-PUCV IMAF (homologous titre 1/2560) cross-reacted strongly with HYV (titre 1/640) and one of the bovine sera reacted strongly to HYV (titre 1/160) but only weakly to PUCV (titre 1/20). These data indicate that HYV is distinct antigenically from PUCV and suggest that at least one of the cattle that tested positive by PUCV G protein ELISA had more likely been infected with HYV.

**Table 1:** **Serum neutralisation tests for PUCV against related ephemeroviruses.**

Viruses	Antisera						
	BEFV	BRMV	PUCV	KIMV	ARV	OBOV	KOTV
BEFV	*1280*	20	–	–	–	–	–
BRMV	80	*40*	–	–	–	–	–
PUCV	–^a^	–	*5120*	–	–	–	–
KIMV	–	–	20	*80*	–	–	–
ARV	–	–	–	–	*640*	–	–
OBOV	–	–	20	–	40	*1280*	–
KOTV	–	–	–	–	–	40	*640*

Homologous serum titres are shown in italic.

^a^A titre of < 1/20 was considered negative.

**Table 2:** **Comparison of PUCV and HYV neutralisation titres for control and selected bovine field sera.**

Serum	Description	Neutralisation titre	
		PUCV	HYV
a-PUCV	PUCV immune mouse ascites fluid (IMAF)	1/2560	1/640
a-BEFV	Bovine serum—vaccinated against BEFV	–^a^	–
164 066	Bovine serum—ELISA-positive to KIMV ELISA-negative to PUCV	–	–
163 862	Bovine serum—weak ELISA-positive to PUCV	–	–
164 934	Bovine serum—strong ELISA-positive to PUCV	1/20	1/160
158 733	Bovine serum—ELISA-positive to PUCV (recent seroconversion)	–	1/20

^a^A titre of < 1/20 was considered negative.

Sera collected in June 2000 from 41 cattle in a sentinel herd at Beatrice Hill Farm were tested for neutralising antibodies to HYV (Additional file 6A). The animals had been recruited as sentinels in December 1999 and January 2000 at the same farm as the bull from which HYV was isolated in February 2000. Of the 41 sera, one tested strongly positive for HYV (titre 1/72), four tested weakly positive (titres 1/20 to 1/40), 10 were questionable (titres 1/10 to 1/20) and the remainder were negative (titre <1/10). BEFV neutralising antibody was also detected in 27 of these 41 sera. Neutralisation tests were conducted on sera collected from the five HYV-positive cattle from January to June 2000, to establish when HYV infection may have occurred in these animals. The results indicated a clear seroconversion in one animal (#54) in March 2000 (titre >1/160) indicating that HYV was still circulating at this time (Additional file 6B). Significantly, all five animals had already seroconverted to BEFV on first sampling in January. Sera collected during 2011 and 2012 from nine cattle in sentinel herds either at Beatrice Hill Farm (two animals) or Berrimah Farm (seven animals) were also tested for HYV neutralising antibodies (Additional file 6C). Of these, four animals, all from Berrimah Farm, showed strong evidence of seroconversion to HYV (titres ≥80), with two seroconverting in May 2012 and one each in February and December 2012 (another animal was positive in November 2012, the only time point tested for this animal). However, three of these four animals also appeared to seroconvert to BEFV at the same time (or shortly after) and clinical cases of BEFV were also recorded in the same herd during this time period, indicating co-circulation of BEFV and HYV.

### Experimental infection of cattle

Four cattle were inoculated intravenously with 10^6.8^ TCID_50_ of the cell culture-adapted strain of PUCV and observed for 3 weeks for clinical signs. Rectal temperatures remained normal (37.9–40.2 °C) throughout and appetite was unaffected. One of the four animals had a mild discharge from the right eye on day 3 and day 4 post-inoculation. Blood collected from each animal pre-inoculation (day 0), on days 1 to 7 post-inoculation and at termination of the experiment all tested negative for PUCV RNA by RT-PCR and negative for antibody to PUCV G protein by ELISA.

## Discussion

In Australia, East Asia and the Middle-East, bovine ephemeral fever is considered to be an important disease of cattle, causing significant production losses in both dairy and beef herds [7–9, 43]. BEFV occurs as a single serotype throughout its geographic range through tropical and sub-tropical regions from South Africa to Japan [3]. Although eight other antigenically related, but distinct, ephemeroviruses had been described prior to this study, none have ever been isolated from animals showing signs of disease or shown to cause ephemeral fever experimentally in cattle, with the notable exception of a single recorded outbreak of KOTV in Nigeria [11, 12] and tentatively the as yet uncharacterised Mavingoni virus in Mayotte Island [13]. Indeed, several of these other ephemeroviruses, such as KIMV and ARV, commonly infect cattle in Australia and elsewhere, with no evidence of association with disease [14, 16]. It is, therefore, quite significant that we report the isolation of a novel ephemerovirus from a moribund bovine displaying severe clinical signs of ephemeral fever.

Unfortunately, there was a lag of several years between the disease event and identification of HYV and, with no remaining infectious blood and cell culture passage yielding only low titre material, we were unable to conduct experimental infections to fulfil River’s postulates. An experimental infection in cattle with closely related PUCV was conducted and failed to show evidence of either infection or disease; however, it is well established that, even for BEFV, cattle are usually not susceptible to infection with cell culture adapted virus [44]. Nevertheless, several factors suggest that HYV may have been responsible for the disease. Firstly, other infections that could have caused similar clinical signs, including TSE, babesiosis and BEFV itself, were specifically excluded by laboratory tests. Secondly, HYV was isolated at the time of clinical disease; ephemerovirus infections in cattle are typically brief and virus isolation from blood is usually only possible at or near the time of peak viraemia. Thirdly, although mortalities due to bovine ephemeral fever are relatively uncommon, the most severe cases usually occur in older, larger cattle; in this case the diseased animal was a mature, 2.5 year-old bull. Fourthly, although co-circulation of BEFV and other ephemeroviruses has been reported previously [13], no isolations of BEFV were made during this period from other cattle located at the farm, suggesting BEFV infections were not occurring in the herd at the time. Serological evidence also indicated that BEFV had already passed through the herd several months previously. Although we cannot conclude unequivocally that HYV was the cause of disease, there is certainly sufficient evidence to suggest that testing of cattle in northern Australia for HYV infection during future outbreaks of ephemeral fever would be worthwhile.

HYV is quite closely related, both antigenically and phylogenetically, to PUCV which was isolated from mosquitoes in Malaysia in 1965. This raises the question as to whether HYV and PUCV should be assigned to different species or, like KIMV and MALV, should be considered geographic variants of the same virus [23]. Currently, demarcation criteria approved by the ICTV require that viruses assigned to different species within the genus *Ephemerovirus* have several of the following characteristics: (A) minimum amino acid sequence divergence of 15% in L; (B) minimum amino acid sequence divergence of 8% in N; (C) can be distinguished in serological tests; and (D) significant differences in genome organisation as evidenced by numbers and locations of ORFs [1]. HYV and PUCV sit marginally outside the criteria A and B (12.3% divergence in L and 4.5% divergence in N), can be distinguished in virus neutralisation tests (criterion C) and differ marginally in genome organisation (criterion D), albeit only in an alternative ORF in the β gene (βx) that may or may not be expressed. However, the relationship between BEFV and BRMV (which are already assigned to separate species) is similar to the relationship between HYV and PUCV in terms of whole genome nucleotide sequence divergence and amino acid sequence divergence in the N, G and L proteins (Additional file 3). In this context, it is useful to consider that there is a maximum of 5.7% amino acid sequence divergence (p-distance) amongst G protein ectodomain sequences of all 149 available BEFV strains isolated in Japan, mainland China, Taiwan, Australia and Israel from 1956 to 2012 [23, 45]. By contrast, G protein ectodomain sequence divergence between this set of BEFV isolates and BRMV is in the range 19.1% and 21.8%. Sequence divergence of the equivalent sequences of HYV and PUCV is 18.0%. This argues strongly for the assignment of HYV and PUCV to two new and separate ephemerovirus species. A similar analysis indicated that the divergence between partial G and P protein sequences of KIMV and MALV fall within the range of genetic diversity displayed by BEFV isolates [23].

Serological data presented here indicate that several cattle in the sentinel herd at Beatrice Hill Farm had developed neutralising antibody to HYV by June 2000 (Additional file 6A), including at least one that seroconverted in February–March when HYV was isolated from the moribund bull (Additional file 6B). The data also indicate that HYV was active at Berrimah Farm (located 50 km north-west of Beatrice Hill Farm) in 2011–2012 (Additional file 6C), suggesting that, like several other ephemeroviruses, HYV is endemic in northern Australia. However, due to antigenic cross-reactivity, we cannot exclude the possibility that PUCV is also present in Australia. BEFV and KIMV have a widespread distribution in Australia, extending from the Northern Territory into Queensland, New South Wales and northern regions of Western Australia [14, 45, 46], reflecting the distribution of potential insect vectors [47]. In addition, the data from Berrimah Farm suggests a correlation between seroconversion for HYV and BEFV for animals B01, B12 and possibly B18. Whether this is due to cross-reactivity between the two viruses, or the cocirculation of both viruses in the herd is unknown. Natural infection of cattle with BEFV, KIMV, BRMV or ARV can induce both homologous and heterologus neutralising antibody responses [48]. However, many of the ephemeroviruses may show similar epidemiology and circulation patterns. Further serological testing should be conducted to determine the extent of the geographic range of HYV across the continent and the degree to which this virus co-circulates with other ephemeroviruses.

The organisation of the HYV genome is similar to those of BEFV, BRMV, KIMV and PUCV, featuring genes encoding the same set of accessory proteins (G_NS_, α1, α2, β and γ) between the G and L genes. Of these, only the BEFV G_NS_ and α1 proteins have been identified definitively in infected cells [49, 50], although polyadenylated transcripts for these and the other accessory genes have been identified for several ephemeroviruses [22, 23, 51]. Due to the poor HYV titres obtained in cell culture, our expression analysis here focussed on PUCV. As reported previously for KIMV, MALV, KOTV and OBOV [22, 23], in addition to the major structural proteins and the G_NS_ protein, several virus-induced protein bands were detected with a relative mobility range of approximately 9–15 kDa. Although we could not identify these specifically as the small PUCV accessory proteins (with calculated molecular weights in the range 13.4–16.6 kDa), there was evidence of expression of their corresponding mRNAs. However, assignment of proteins based only on relative migration in polyacrylamide gels is problematic.

Numerous ephemeroviruses have been identified previously in Australian cattle, but this is the first time that a non-BEFV ephemerovirus has been tentatively linked to clinical disease. Although no infection or disease resulted from the experimental inoculation of cattle with the closely related PUCV (possibly due to cell culture adaptation), differences in clinical outcomes between pairs of closely related ephemeroviruses are well known. Both known agents of bovine ephemeral fever, BEFV and KOTV, are closely related to viruses that do no cause disease (BRMV and KOOLV, respectively [15, 17]). In addition, bovine ephemeral fever-like disease has been observed in Australia in the absence of BEFV seroconversion, indicating the likely involvement of another pathogen [2]. As an ephemerovirus, HYV is certainly a potential candidate. Although it has only been tentatively linked to a single case of disease so far, the serological evidence suggests that this virus regularly infects Australian cattle. This indicates the need for the further study of HYV, to establish its distribution, its impact on the evolution of BEFV and, most importantly, whether it truly is an agent of disease.

## Supplementary information

**Additional file 1. Primers used in the study.** Details of primers used for RACE for HYV and PUCV and details of primers used for transcription profiling on PUCV.

**Additional file 2. Initial phylogenetic analysis of isolate V4816 (also referred to as DPP4816).** A Neighbour-Joining phylogenetic tree inferred from a Clustal W alignment of the deduced amino acid sequence of the 100 nt amplicon generated from isolate DPP4816 (using an RT-PCR targeting the L genes of ephemeroviruses and several related rhabdoviruses) and the corresponding L protein sequences of Puchong virus (PUCV; MH507505), Kimberley virus (KIMV; NC_025396), Malakal virus (MALV; NC_025400), Berrimah virus (BRMV; NC_025358), bovine ephemeral fever virus (BEFV; NC_002526), Yata virus (YATV; NC_028241), kotonkan virus (KOTV; NC_017714), Koolpinyah virus (KOOLV; NC_028239), Adelaide River virus (ARV; NC_028246), Obodhiang virus (OBOV; NC_017685), Wongabel virus (WONV; NC_011639), Fukuoka virus (FUKV; NC_034454) and Tibrogargan virus (TIBV; NC_020804). The alignment and tree were generated in MEGA version 7.0.18 using default parameters.

**Additional file 3. Sequence identities amongst ephemeroviruses.** Comparison of nucleotide and amino acid sequence identities of HYV and PUCV to other related ephemeroviruses.

**Additional file 4. Comparison of ephemerovirus G and G**_**NS**_**proteins.** A Clustal W amino acid sequence alignment of the G and G_NS_ proteins of BEFV, HYV and PUCV. The alignment was generated in MEGA version 7.0.18 using default parameters and adjusted following visual inspection. Identical (*), strongly conserved (:) and weakly conserved (.) amino acids are indicated. Predicted signal peptides) in the N-terminal domains and predicted transmembrane domains in the C-terminal domains are shaded in grey. Predicted N-glycosylation sites are underlined. Conserved cysteine residues in the ectodomains are shaded in black. Twelve cysteine residues (C_I_–C_XII_) in the BEFV G also occur in the G protein of vesicular stomatitis Indiana virus in which they form six disulphide bridges indicated by dotted lines (see text). Six additional cysteine residues (a–f) occur in the BEFV G protein and have been predicted to form three additional disulphide bridges (see text). The figure illustrated similarities in the structure of the G and G_NS_ proteins of BEFV, HYV and PUCV.

**Additional file 5. Clustal W amino acid sequence alignments of the small accessory proteins of HYV, PUCV and BEFV.** A) α1 proteins. Predicted transmembrane domains are shaded (grey). Large aromatic residues in the N-terminal domains (underlined) and basic residues in the C-terminal domains (bold) are characteristic of class 1a viroporins. B) α2 proteins which are each encoded in a second consecutive ORF within the α gene. C) β proteins. D) γ proteins. Identical (*), strongly conserved (:) and weakly conserved (.) amino acids are indicated.

**Additional file 6. Sero-neutralisation test results.** Neutralising antibody titres to HYV in sera from selected sentinel cattle from the Northern Territory, Australia.

## Data Availability

The datasets analysed during the current study available from the corresponding author on reasonable request.

## Abbreviations

ARV: Adelaide River virus
BEF: bovine ephemeral fever
BEFV: bovine ephemeral fever virus
BHK-BSR: baby hamster kidney cells
BME: Basal Medium Eagle
BRMV: Berrimah virus
C6–36: *Aedes albopictus* cells
hpi: hours post-infection
HYV: Hayes Yard virus
IMAF: immune mouse ascetic fluids
KIMV: Kimberley virus
KOOLV: Koolpinyah virus
KOTV: Kotonkon virus
MALV: Malakal virus
MOI: multiplicity of infection
MVGV: Mavingoni virus
Mr: molecular mass
NGS: next generation sequencing
OBOV: Obodhiang virus
PUCV: Puchong virus
RACE: rapid amplification of cDNA ends
SISPA: sequence-independent single primer amplification
TSE: transmissible spongiform encephalopathy
TI: transcription initiation
TTP: transcription termination/polyadenylation
TURBS: termination upstream ribosome binding sites
WONV: Wongabel virus
YATV: Yata virus
