# Supplementary material for: Hayes Yard virus: a novel ephemerovirus isolated from a bull with severe clinical signs of bovine ephemeral fever is most closely related to Puchong virus
Source: Vet Res. 2020 Apr 29;51:58. doi: 10.1186/s13567-020-00781-1 (PMC7191811; doi:10.1186/s13567-020-00781-1)
Supplement: Supplementary file 1 — Additional file 1. Primers used in the study. Details of primers used for RACE for HYV and PUCV and details of primers used for transcription profiling on PUCV. [file 13567_2020_781_MOESM1_ESM.docx]

Details of primers used for RACE.

| **Virus** | **Direction** | **Primer ID** | **Sequence** | **Position** |
| --- | --- | --- | --- | --- |
| PUCV | 3’ | PUCV_3.1 | GCTTCAAGGCAATGATCTCC | 113 |
|  |  | PUCV_3.2 | CCTTGAGGGATACGGAGAGTT | 198 |
|  |  | PUCV_3.3 | CATACATAATTCTCGGGCAG | 236 |
|  | 5’ | PUCV_5.1 | GCATTACGACCCAAGATTTAG | 14808 |
|  |  | PUCV_5.2 | CGTTGGTATGAGAGATTCAGG | 14695 |
|  |  | PUCV_5.3 | ATGGTGAGTACCGGGAAGTGT | 14602 |
| HYV | 3’ | HYV3R_1 | GTCTACTGGAAACCGACTGA | 473 |
|  |  | HYV3R_2 | CCTTATCGAAACCACACTTG | 350 |
|  |  | HYV3R_3 | GGTTGTAATGCCTATGGTGTTCC | 199 |
|  | 5’ | HYV5F_1 | GTCCTGACTTGTTGATTGAGC | 14319 |
|  |  | HYV5F_2 | GAGCCTAGCATTGAGACTGAGCCTGG | 14566 |
|  |  | HYV5F_3 | GGAACTGGATACGATGGTAC | 14778 |

Details of primers used for transcription profiling on PUCV.

| **Target gene** | **Primer ID** | **Sequence** | **Size (bp)** |
| --- | --- | --- | --- |
| N | PUCV_1046_N | ACAAGAGGACAGGAAGTATG | 264 |
| P | PUCV_598_P | TATGGGTAGTCTGAAATCTG | 240 |
| M | PUCV_436_M | CCATCCGAAAGAATTAGAAG | 294 |
| G | PUCV_1746_G | CAAGGTCAGAAGAGGATAGG | 235 |
| G_NS_ | PUCV_1542_GNS | ATGGAGTAGAGGAGGTACAG | 217 |
| α1 | PUCV_56_A1 | AGATAGAGGGAGTGAGATTC | 311 (660 if read-through) |
| α2 | PUCV_6_A2 | CATCAGGAGTGGGAGGAAAC | 357 |
| β | PUCV_66_B | GCGTGCTTTATTAGGGCTTC | 385 |
| γ | PUCV_43_GM | TGTTGATAACGGTATGTCTG | 310 |
| L | PUCV_6156_L | GCCTTCAGGGAAGATAGAAC | 308 |
