# Supplementary figures and images for: Hayes Yard virus: a novel ephemerovirus isolated from a bull with severe clinical signs of bovine ephemeral fever is most closely related to Puchong virus
Source: Vet Res. 2020 Apr 29;51:58. doi: 10.1186/s13567-020-00781-1 (PMC7191811; doi:10.1186/s13567-020-00781-1)

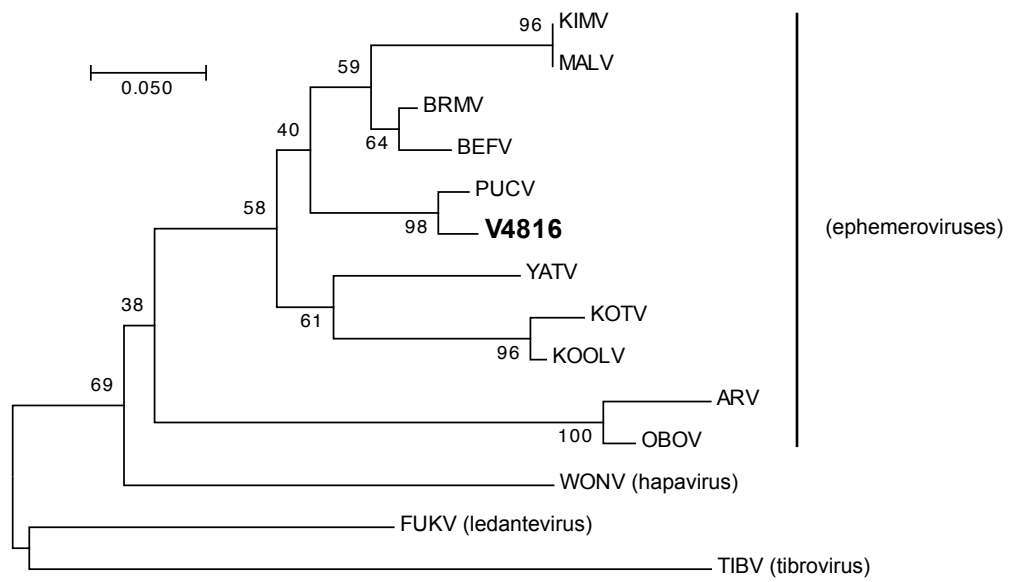

Supplement: Supplementary file 2 — Additional file 2. Initial phylogenetic analysis of isolate V4816 (also referred to as DPP4816). A Neighbour-Joining phylogenetic tree inferred from a Clustal W alignment of the deduced amino acid sequence of the 100 nt amplicon generated from isolate DPP4816 (using an RT-PCR targeting the L genes of ephemeroviruses and several related rhabdoviruses) and the corresponding L protein sequences of Puchong virus (PUCV; MH507505), Kimberley virus (KIMV; NC_025396), Malakal virus (MALV; NC_025400), Berrimah virus (BRMV; NC_025358), bovine ephemeral fever virus (BEFV; NC_002526), Yata virus (YATV; NC_028241), kotonkan virus (KOTV; NC_017714), Koolpinyah virus (KOOLV; NC_028239), Adelaide River virus (ARV; NC_028246), Obodhiang virus (OBOV; NC_017685), Wongabel virus (WONV; NC_011639), Fukuoka virus (FUKV; NC_034454) and Tibrogargan virus (TIBV; NC_020804). The alignment and tree were generated in MEGA version 7.0.18 using default parameters. [file 13567_2020_781_MOESM2_ESM.pdf]
