# Supplementary material for: Hayes Yard virus: a novel ephemerovirus isolated from a bull with severe clinical signs of bovine ephemeral fever is most closely related to Puchong virus
Source: Vet Res. 2020 Apr 29;51:58. doi: 10.1186/s13567-020-00781-1 (PMC7191811; doi:10.1186/s13567-020-00781-1)
Supplement: Supplementary file 3 — Additional file 3. Sequence identities amongst ephemeroviruses. Comparison of nucleotide and amino acid sequence identities of HYV and PUCV to other related ephemeroviruses. [file 13567_2020_781_MOESM3_ESM.docx]

Nucleotide sequence identities (p-distances) amongst ephemerovirus full-length genomes.

|  | BEFV | BRMV | KIMV | MALV | PUCV | HYV |
| --- | --- | --- | --- | --- | --- | --- |
| BEFV |  |  |  |  |  |  |
| BRMV | 67.0 |  |  |  |  |  |
| KIMV | 44.7 | 44.0 |  |  |  |  |
| MALV | 44.9 | 44.2 | 90.7* |  |  |  |
| PUCV | 40.0 | 39.7 | 38.9 | 39.4 |  |  |
| HYV | 40.6 | 40.5 | 40.2 | 39.6 | 68.8 |  |

* KIMV and MALV are considered to be geographic variants of the same virus (species *Kimberley ephemerovirus*).

Amino acid sequence identities (p-distances) of ephemerovirus N proteins.

|  | BEFV | BRMV | KIMV | MALV | PUCV | HYV | ARV | OBOV | KOTV | KOOLV | YATV |
| --- | --- | --- | --- | --- | --- | --- | --- | --- | --- | --- | --- |
| BEFV |  |  |  |  |  |  |  |  |  |  |  |
| BRMV | 91.7 |  |  |  |  |  |  |  |  |  |  |
| KIMV | 78.3 | 78.0 |  |  |  |  |  |  |  |  |  |
| MALV | 78.3 | 78.3 | 98.6* |  |  |  |  |  |  |  |  |
| PUCV | 76.4 | 78.0 | 74.2 | 75.2 |  |  |  |  |  |  |  |
| HYV | 76.1 | 77.5 | 74.5 | 75.2 | 95.5 |  |  |  |  |  |  |
| ARV | 49.4 | 49.6 | 50.8 | 51.3 | 50.1 | 50.4 |  |  |  |  |  |
| OBOV | 50.6 | 50.1 | 52.0 | 52.5 | 51.1 | 51.3 | 87.2 |  |  |  |  |
| KOTV | 51.3 | 51.5 | 53.2 | 52.7 | 53.0 | 52.2 | 48.0 | 46.3 |  |  |  |
| KOOLV | 51.8 | 51.5 | 52.7 | 52.5 | 52.5 | 51.8 | 47.3 | 46.6 | 92.7 |  |  |
| YATV | 45.6 | 46.1 | 47.8 | 48.0 | 45.4 | 46.1 | 41.6 | 39.2 | 51.5 | 50.8 |  |

* KIMV and MALV are considered to be geographic variants of the same virus (species *Kimberley ephemerovirus*).

Amino acid sequence identities (p-distances) of ephemerovirus G proteins.

|  | BEFV | BRMV | KIMV | MALV | PUCV | HYV | ARV | OBOV | KOTV | KOOLV | YATV |
| --- | --- | --- | --- | --- | --- | --- | --- | --- | --- | --- | --- |
| BEFV |  |  |  |  |  |  |  |  |  |  |  |
| BRMV | 79.0 |  |  |  |  |  |  |  |  |  |  |
| KIMV | 50.0 | 49.5 |  |  |  |  |  |  |  |  |  |
| MALV | 50.2 | 50.2 | 95.6* |  |  |  |  |  |  |  |  |
| PUCV | 50.9 | 50.0 | 47.7 | 46.8 |  |  |  |  |  |  |  |
| HYV | 50.2 | 49.6 | 48.9 | 48.2 | 80.8 |  |  |  |  |  |  |
| ARV | 30.3 | 30.6 | 27.5 | 27.5 | 29.6 | 29.2 |  |  |  |  |  |
| OBOV | 30.6 | 30.6 | 29.0 | 29.4 | 29.9 | 29.6 | 73.1 |  |  |  |  |
| KOTV | 30.1 | 29.6 | 31.9 | 32.0 | 29.4 | 28.7 | 27.8 | 27.1 |  |  |  |
| KOOLV | 31.0 | 29.6 | 31.7 | 31.7 | 27.6 | 28.2 | 27.8 | 28.3 | 81.7 |  |  |
| YATV | 30.1 | 30.5 | 30.8 | 31.2 | 30.1 | 30.6 | 29.4 | 31.2 | 40.1 | 39.6 |  |

* KIMV and MALV are considered to be geographic variants of the same virus (species *Kimberley ephemerovirus*).

Amino acid sequence identities (p-distances) of ephemerovirus L proteins.

|  | BEFV | BRMV | KIMV | MALV | PUCV | HYV | ARV | OBOV | KOTV | KOOLV | YATV |
| --- | --- | --- | --- | --- | --- | --- | --- | --- | --- | --- | --- |
| BEFV |  |  |  |  |  |  |  |  |  |  |  |
| BRMV | 85.5 |  |  |  |  |  |  |  |  |  |  |
| KIMV | 65.8 | 66.0 |  |  |  |  |  |  |  |  |  |
| MALV | 65.7 | 65.9 | 96.9* |  |  |  |  |  |  |  |  |
| PUCV | 64.7 | 64.7 | 64.5 | 64.4 |  |  |  |  |  |  |  |
| HYV | 64.6 | 65.3 | 64.5 | 64.7 | 87.7 |  |  |  |  |  |  |
| ARV | 50.2 | 49.7 | 49.3 | 49.3 | 48.1 | 48.2 |  |  |  |  |  |
| OBOV | 50.5 | 49.8 | 50.3 | 50.5 | 49.1 | 49.0 | 79.8 |  |  |  |  |
| KOTV | 51.6 | 52.3 | 51.1 | 51.0 | 50.6 | 50.8 | 48.5 | 48.5 |  |  |  |
| KOOLV | 51.8 | 51.7 | 51.3 | 51.3 | 50.4 | 50.5 | 48.2 | 48.8 | 85.0 |  |  |
| YATV | 51.2 | 51.6 | 51.7 | 51.8 | 51.1 | 51.4 | 47.8 | 47.5 | 59.6 | 59.8 |  |

* KIMV and MALV are considered to be geographic variants of the same virus (species *Kimberley ephemerovirus*).
