# Supplementary material for: Hayes Yard virus: a novel ephemerovirus isolated from a bull with severe clinical signs of bovine ephemeral fever is most closely related to Puchong virus
Source: Vet Res. 2020 Apr 29;51:58. doi: 10.1186/s13567-020-00781-1 (PMC7191811; doi:10.1186/s13567-020-00781-1)
Supplement: Supplementary file 4 — Additional file 4. Comparison of ephemerovirus G and GNSproteins. A Clustal W amino acid sequence alignment of the G and GNS proteins of BEFV, HYV and PUCV. The alignment was generated in MEGA version 7.0.18 using default parameters and adjusted following visual inspection. Identical (*), strongly conserved (:) and weakly conserved (.) amino acids are indicated. Predicted signal peptides) in the N-terminal domains and predicted transmembrane domains in the C-terminal domains are shaded in grey. Predicted N-glycosylation sites are underlined. Conserved cysteine residues in the ectodomains are shaded in black. Twelve cysteine residues (CI–CXII) in the BEFV G also occur in the G protein of vesicular stomatitis Indiana virus in which they form six disulphide bridges indicated by dotted lines (see text). Six additional cysteine residues (a–f) occur in the BEFV G protein and have been predicted to form three additional disulphide bridges (see text). The figure illustrated similarities in the structure of the G and GNS proteins of BEFV, HYV and PUCV. [file 13567_2020_781_MOESM4_ESM.docx]

**a** **CI** **b** **CII**

BEFV_G MFK------VLIITLLVNKIHLE-KIYNVPVNCGELHPVKAHEIKCPQRLNELSLQAHHNLAK-DEHYNKICRPQLKDDAHLEGFIC

HYV_G MISKILCWMALGQCLRVTRAETESRVFNVPVNCMEERPIKPTEIVCPKRYNELSLEAHHTLIEGEEKIEQICRPALRDDDHVDGYIC

PUCV_G MISKIQSCIIFGLCSRVFSADIGSKVFNVPVNCVGEKPIKPTEITCPKRYNELSLEAHHTLIEGDEKVEQICRPALRDDDHVEGYVC

BEFV_Gns MFL-----QLFNIVLIYGVRTSQSTWINYPENCTSISLQDGLRELCGG--DQLMNIRNQLLDDTYKEIGEICTPNYSMEKKSEGYRC

HYV_Gns MWI-----YLISFSMV--VSICEGYWVNYPTECKDLNSEEAIRDLCGG--NSLINIRNTLLDDSWEKIGEICTPDNSKSKRVRGFFC

PUCV_Gns MWA-----LLIRQLLF--LKITNAYWVNYPKECKDLNSESAIEDLCGG--DSLINIRNTLLDDSYEKIGEICTPDNTKSRRVRGYYC

* : * * :* . . * :.* : * . :. :** * . : *: *

**CIII** **CIV** **CV**

BEFV_G RKQRWITKCSETWYFSTSIEYQILEVIPEYSGCTDAVKKLDQGALI------PPYYPPAGCFWNTEMNQEIEFYVLIQHKPFLNPYD

HYV_G REQHWETECEETWYFATVINYHIRETIPSPGDCISAVKQFKDGVLI------PPYYPPAGCFWNTKIKETIKFMVLTRHKSILNPMD

PUCV_G REQHWETECEETWYFATVINYHIREKPPSLSDCITAVKQYKDGILI------PPYYPPAGCFWNTKIKETIKFMVLIKHRSILNPID

BEFV_Gns ASIKKKVICKMLENFDHEVTYISESHPIDKAKCHELIINKDLLNNIE-----EPYYPPPKCDSSKSSVSELEFIKLINYDVILDPVG

HYV_Gns TAVTKKTTCKVLENLDNKVTYETTIRKVGREECYNHIQDLNLKTEEESQKNLAPFYPPPKCELGKEETVVKTFMVLDETEINLNPLD

PUCV_Gns TEITKKTTCKVLGNFDNKVTYETTIRKVGRKECYSHIQNLNHRPDYESQESVAPFYLPPKCEIGDGKTVSKTFMILEETEINLNPTD

. *. : : * * : . . *:* *. * . * * . *:* .

**CVI** **CVII** **CVIII** **c**

BEFV_G NLIYDSRFLTPCTINDSKTKGCPLKDITGTWIPDVRVEEISEHCNNKHWECITVK--------------SFRSELNDKERLWEAPDI

HYV_G NLVHDSQFINPCDLKKTQKTGCKLKDVTGLWIPEIEIGLSSEHCSRKHWECIGIK--------------SYKSELDEKIRIWEAPEI

PUCV_G NLVHDTQFINPCDLKGAQKDGCKLKDITGLWIPELENGLSSEHCSKKHWECIGIK--------------SYKSEVDEKTRIWEAPEI

BEFV_Gns FQNEDNYLFQ--------------FDKTNPIPID--YIYQSEFCQSKNWICHGDKSYIPLEIFKGDNQASIRLELIKLSIIYDS-NF

HYV_Gns FEKEDLYLFS--------------LENNRSIIENDLLVERSEYCKLSNWKCHGRKNHIPLEIFKDDDQVSIRLELLKLNILYDS-EY

PUCV_Gns LEKEDLYLFS--------------MK-NRSIIEDDILVERSEYCKLTNWKCHGKKNHIPLEIFKDDDQVSIRLELLKLNIIYDS-EY

* . . . : **.*. .:* * * * : *: : :::: :

**CIX** **CX** **CXI** **CXII**

BEFV_G GLVHVNKGCLSTFCGKNGIIFEDGEWWSIENQTESDFQNFKIEKCKGKKP-----GFRMHTDRTEFEELDIKAELEHERCLNTISKI

HYV_G GIINITKSCKQSFCGFKGVVFDDGEWWGYANTTEDELIKAHVSDCKDRKP-----GIRVHNDHTDYETYDIRAEMENERCQNTISKI

PUCV_G GIINITKSCKQSFCGYRGVVFDDGEWWGYANETEAELIDAHIPTCSGKKP-----GIRVHNDHTDYETYDIRAEMENERCQNTISKI

BEFV_Gns GELPIRDACRLHYCGKPAIKLFNGAIIKIKE----SPIVLGLPSCNRSRIEMPETNLAKKRYSNVGPVLLTTLNKRFELCKKIKKNL

HYV_Gns GELPLYNSCKMTFCGKEVVRTEGGAIILLRH----NGIVEKTRECDRRERNSPIQDLSRKVFKTIGPILLSTLYKRHDLCRKIKDNL

PUCV_Gns GELPLYNSCKMTFCGREVVKTENGAILLLRH----KGIVEKTRVCNKEEKVGPIQDLSRKVFKTIGPILLSTLYKRHELCRRIKNNL

* : : ..* :** : .* . *. .: : . . : * . .::

**d e f**

BEFV_G LNKENINTLDMSYLAPTRPGRDYAYLFEQTSWQEKLCLSLPDSGRVSKDCNIDWRTSTRGGMVKKNHYGIGSYKRAWCEY-------

HYV_G LNSEPINTVDMSYLSPTRPGRDYAYRFKQVNWTETFCLRWVQTG-LSKDCNKFWKYSDRGGKVTKDHVGIGEYTRALCEF-------

PUCV_G LNSEPINTIDMSYLSPTRPGRDFAYRFKQVNWTETFCLRWAESG-LIKDCRKHWKLSDRGGKVTKEHVGIGGYTRALCEF-------

BEFV_Gns ELKQPIPINNLHYLAPFEPGKHPALVYRLVSTTINQS-------------------------LRNKVVPVSMLSMSMCEYITGQIIE

HYV_Gns KNGRPVPFENLNYINPFEPGWHPAAHYVRIKTSINGA-------------------------LRGRLIEATKLQFKSCNYEIGDAHP

PUCV_Gns KNGKPVPLENLNYINPFEPGWHPAAHYVRIKTSMSGA-------------------------IRGRLIEATKLQFKSCNYEIGDAQP

. : :: *: * .** . * : . : : *::

BEFV_G ----------RPFVDKNEDGYIDIQELNGHNMSGNHAILETAPAGGSS-GNRLNVTLNGMIFVEPT-----KLYLHTKSLYEGIEDY

HYV_G ----------RPIIDKDGDGYISKEELGKHNMSTSFSLMRLDKKGGNQGADSIEVGFNGIVRIPKSEKDGEKYMVRMTSVYDGLEQR

PUCV_G ----------RPIVDQDGDGYISKTELGRHNMSAEFSSMKLGTRSSNQPVDSLDVGFNGIVKVTDDRTREEKYMVRTTSVYDGLEQR

BEFV_Gns DGIKRNLTDEDTVIILANNKEIKWKDLKGR--ENWYQEQANPNIIDKNPDHLSYYWYNGVMRREDKFTYPSRYILQTLKKIYTDTER

HYV_Gns ----KNGTTNFT-ITFGQGKMITHDNIASR--EGWVVESIDPEQDQNFTKGRVRVWYNGVMERDNELFFPTTFLIKKFESIYKDKVM

PUCV_Gns ----LNATTNLE-IRFGQGKVVKYENITQR--EGWTVETTDPDQTQNLTDGNIKVWYNGVIMRDNKIFLPTTFLINKFESIYKDKVM

: . : :: : . . **:: :. .

BEFV_G QKLIKFEVMEYDNVEENLIR-YEEDEKFKPVNLNPHEKSQINRTDIVREIQKGGKKVLSAVVGWFTSTAKAVRWTIWAVGAIVTTYA

HYV_G NRLIKFEVLEFEDVVAKYQGEGNYDVNDKVVELIPDNQKNISRTDFVRTIANGGKEIISGIVGWFTGTAKIIRWTIWAIGALVTTYA

PUCV_G NRLMKFEVLEFEDVVSKYQGEGHYDVKGKSIDLTPDEQKSINRTDFVKTIANGGKEIVSGIVGWFTGTAKLVRWTIWAVGALVTTYA

BEFV_Gns ESRISFFKFRLERNITKTEVIKFRDIEES---SDQDHSQSVNKTLEGDDYWNWVEETTSDKNKTDGSRGDEK-QTIQNKEYWNEESS

HYV_Gns ISLDGTDSLWVKDNRT--------EIIVK---LEKDTSTKTNLTEDEWSNLNHSVHKVGVEDGTVIMRSNET-ITIENKSYFTEDFS

PUCV_Gns INLEGTDSIWIKDNVT--------EIVVR---LEKDQSVKINKSEIIWDNSTDNILQEENGVEEVQIKANET-ITIGNKSYFTEDFS

: . : . . . . : . .. ** :

BEFV_G IYKLYKMVKSNSSHSKHREADLEGLQSTTK----ENMRVEKNDKNYQDLELGLYEEI--------RSIKGGSKQTGDDRFFDH

HYV_G IYKLHKLVRGKNKQESQNYQGEQGGQTESKPKWFKRGRREETPKKSIRLENPLYEEIDIDDNYSLRHKDSGRLKLKKDGFFDV

PUCV_G IYKLHKLVRGKPRSEEDRSQAEDRAEENKRSSWFNSGKKNDLRKNPLKHDNPLYEEIDLEDTYSMRKKEGWKTDRTRDNFFDV

BEFV_Gns IWGISTIITVLGIYYIYRKNRREK----------IFLNMKHRVQRFFKLDY--------------------------------

HYV_Gns TWSIYTILSVLGLWVLYRKGVSKG----------NKVRVRKFIQRFFKLDYRQ------------------------------

PUCV_Gns TWSVYTILSVLGLWILYRRGVSKG----------NKVRVRNLFQKFFKLDYR-------------------------------

: : .:: . . . .. :. :
