# Supplementary material for: Hayes Yard virus: a novel ephemerovirus isolated from a bull with severe clinical signs of bovine ephemeral fever is most closely related to Puchong virus
Source: Vet Res. 2020 Apr 29;51:58. doi: 10.1186/s13567-020-00781-1 (PMC7191811; doi:10.1186/s13567-020-00781-1)
Supplement: Supplementary file 5 — Additional file 5. Clustal W amino acid sequence alignments of the small accessory proteins of HYV, PUCV and BEFV. A) α1 proteins. Predicted transmembrane domains are shaded (grey). Large aromatic residues in the N-terminal domains (underlined) and basic residues in the C-terminal domains (bold) are characteristic of class 1a viroporins. B) α2 proteins which are each encoded in a second consecutive ORF within the α gene. C) β proteins. D) γ proteins. Identical (*), strongly conserved (:) and weakly conserved (.) amino acids are indicated. [file 13567_2020_781_MOESM5_ESM.docx]

**A**

HYV alpha1 MDT-PFRKLWENIKNWGEERGNEIKNWWHPLEWRLKI-FGIVVLLLVGSVVLYKVG**K**LLFYCINCLY**K**GGN**K**I**R**ILT**KK**I**R**T**KRKR**IV

PUCV alpha1 MDK-PFRKLWEDIKRWGEDRGSEIQNWWGLLEWRLKI-FGIIILLLVGSVVLYKIG**K**ILF**H**CANCLY**K**GG**K**SI**K**GLT**RR**I**R**N**RRK**TIL

BEFV alpha1 MEKGLLSNFWNDFKRWSEDRKVEIVIWWSNLESKVRLGFWIILIILLG-ILAIRIAI**K**VYQCV**K**FTNQGV**KK**I**KR**II-----**KRKR**SI

*:. : ::*:::*.*.*:* ** ** ** :::: * *::::*:* :: ::. :: * : :* :.*: : :: *.:** :

HYV alpha1 P**HR**F**KRKK**IN**KH**GWN**K**EMSSQ**R**ENVY**K**GWEET**K**MY

PUCV alpha1 P**KK**L**K**T**KR**FA**KH**GG**K**A**K**ILNL**R**EDV**H**QEWEET**K**MY

BEFV alpha1 -**KKYRK**T----------------------------

:: : .

**B**

HYV alpha2 MGGIKKCLLSGKMFIRAGRKPKCTKRASIHKLELWSAISDGLQILEKPNKIYNSERREIEICVSRKINLINNWISKQELDG-YGVEI

PUCV alpha2 MGGKQKYLICGKMFIRSGRKPKCTRRTSIHKLEIWSAILDGLQKLEKQNKISNSEKTEIEICVSRNINLFDNWITKQVLEG-YGLEI

BEFV alpha2 MFG--------YMEIS-VRVEIGKQNSRIHKLELWKLMEEGLHTLMKEEKLDIMLKEEANFGFCRWLNTRGNWLYLEDMRKPILIEF

* * * * * .:.: *****:*. : :**: * * :*: : * :: ..* :* .**: : : :*:

HYV alpha2 ELYSEQELESSQREVICTQVCEPDNYLLGEYKNYILELHYTIIKLE

PUCV alpha2 EIYSEQEFDGKEREVTCNQVCEPDNYLLGEYKNYIMEINYSTILLK

BEFV alpha2 QNFFN-CLNYPSR-VY-KLTVQNNDYKLGSIRDLRIKLFFF-----

: : : :: .* * . . : ::* **. :: ::: :

**C**

HYV_beta MDFSRCKLSLQVMNFKALDLDRRALLGLLIVKSIRTLYRSNQMITRLAGILVPMATLNGEFVVRNDMKKGHWIFVGEAYSALDTMDL

PUCV_beta MDFSRCKLSLQVMNFKALDLDRRALLGLLVVKSIKTLYRSNQMITRLSEILIPMATLNGEFIVRNDMKKGHWIFVGEAYSALDTMDL

BEFV_beta MDFIRCHVAMQIINFKALEIDKRSLLGILVIKNIKNLHRSNQLLTRLSDLMVPSVIHNGEFVMRNDKSDKLWIFVGESWASLDLEDL

*** **::::*::*****::*:*:***:*::*.*:.*:****::***: :::* . ****::*** .. ******::::** **

HYV_beta EGVREQTTHIEKVMPLLINGEEYGAIDLSIKVELGTLRFIKRKGDGDICEIPRRVRILE-

PUCV_beta DGVRDRVTKIEKVLPLLINGEEYGAIDLSINVEVGTLSFIKRKGEGDLCEIPRKVRVLT-

BEFV_beta NGVRDNVFNISKTVPLLIQGEEYGVIDLSIKVEPRGLRFLKRSSEIDICDIPKKVRVVPT

:***:.. :*.*.:****:*****.*****:** * *:**..: *:*:**::**::

**D**

HYV_gamma MDLKFRCLIRNVDNGMSGELIVIEVLNVIRARFMNLPNFDLLDIRSSMFDQESNNPWVYVFGKIHVSGVMGRFIGKRLVNRGSFKIK

PUCV_gamma MDLKFRCLIRNVDNGMSGELIVVEVLNVLRTGFMNLPNFDLLDIRSSMFDQESNNPWVYVFGRVHVSGIMSRFIGRKMVTRGSYKIK

BEFV_gamma MDLKFRCLIKNVADGRAGEIIVEECLEIIEQKYLRLMTIDLKEIRSSMFDQESN-PWVYVFGKIYISGLMGRAIGKRMVKRGTYRIK

*********:** :* :**:** * *:::. ::.* .:** :*********** *******::::**:*.* **:::*.**:::**

HYV_gamma KEWISNHFEDVHIHFYYDMGRIFN----

PUCV_gamma KEWISNHFEDVHIHFYSDIGKIYR----

BEFV_gamma EGELINHFEGVHIHFYKDIEKIFHSIRV

: : ****.****** *: :*:.
