# Supplementary material for: Hayes Yard virus: a novel ephemerovirus isolated from a bull with severe clinical signs of bovine ephemeral fever is most closely related to Puchong virus
Source: Vet Res. 2020 Apr 29;51:58. doi: 10.1186/s13567-020-00781-1 (PMC7191811; doi:10.1186/s13567-020-00781-1)
Supplement: Supplementary file 6 — Additional file 6. Sero-neutralisation test results. Neutralising antibody titres to HYV in sera from selected sentinel cattle from the Northern Territory, Australia. [file 13567_2020_781_MOESM6_ESM.docx]

Additional file 6A: Neutralising antibody titres to HYV in sera collected in June 2000 from sentinel cattle recruited at Beatrice Hill Farm on 9 December 1999 or 27 January 2000. BEFV neutralising antibodies were screened at 1:10 only.

| **Sentinel animal number** | **Serum collection date** | **HYV neutralising antibody titre (if ≥10)** | **BEFV neutralising antibodies (screened at 1:10 only)** |
| --- | --- | --- | --- |
| 1 | 1 Jun 2000 | negative | negative |
| 2 | 6 Jun 2000 | negative | + |
| 3 | 6 Jun 2000 | negative | + |
| 4 | 1 Jun 2000 | negative | + |
| 5 | 1 Jun 2000 | negative | negative |
| 6 | 1 Jun 2000 | negative | negative |
| 7 | 6 Jun 2000 | negative | + |
| 8 | 6 Jun 2000 | 18 | negative |
| 9* | 1 Jun 2000 | 72 | + |
| 10 | 1 Jun 2000 | 12 | + |
| 11 | 6 Jun 2000 | 16 | + |
| 12 | 1 Jun 2000 | negative | + |
| 13 | 1 Jun 2000 | 16 | negative |
| 14 | 6 Jun 2000 | 18 | + |
| 15 | 1 Jun 2000 | negative | negative |
| 16 | 6 Jun 2000 | negative | + |
| 17 | 1 Jun 2000 | negative | + |
| 18 | 6 Jun 2000 | negative | negative |
| 19 | 1 Jun 2000 | negative | negative |
| 20 | 6 Jun 2000 | negative | negative |
| 21 | 6 Jun 2000 | negative | + |
| 22 | 6 Jun 2000 | negative | + |
| 23 | 1 Jun 2000 | negative | negative |
| 24 | 6 Jun 2000 | 16 | + |
| 25 | 6 Jun 2000 | 18 | + |
| 26 | 6 Jun 2000 | negative | + |
| 27 | 6 Jun 2000 | negative | + |
| 28 | 6 Jun 2000 | negative | + |
| 52* | 1 Jun 2000 | 20 | + |
| 53 | 1 Jun 2000 | 16 | + |
| 54* | 1 Jun 2000 | 24 | + |
| 55 | 1 Jun 2000 | 16 | + |
| 57 | 1 Jun 2000 | negative | + |
| 58* | 1 Jun 2000 | 40 | + |
| 61 | 1 Jun 2000 | negative | negative |
| 62 | 1 Jun 2000 | negative | + |
| 63 | 1 Jun 2000 | negative | + |
| 66* | 1 Jun 2000 | 20 | + |
| 68 | 1 Jun 2000 | negative | negative |
| 69 | 1 Jun 2000 | negative | negative |
| 71 | 1 Jun 2000 | 12 | negative |

* Animal selected for further testing (see Additional file 6B).

Additional file 6B: Neutralising antibody titres to HYV in sera collected monthly from five sentinel cattle recruited at Beatrice Hill Farm on 27 January 2000. BEFV neutralising antibodies were screened at 1:10 only.

| **Sentinel animal number** | **Neutralising antibody** | | | | | | | | | |
| --- | --- | --- | --- | --- | --- | --- | --- | --- | --- | --- |
|  | 21 Jan 2000 | | 24 Feb 2000 | | 23 Mar 2000 | | 27 Apr 2000 | | 1 Jun 2000 | |
|  | HYV | BEFV | HYV | BEFV | HYV | BEFV | HYV | BEFV | HYV | BEFV |
| 9 | 64 | + | 32 | + | 20 | + | 24 | + | 72 | + |
| 52 | 10 | + | 18 | + | 18 | + | 32 | + | 20 | + |
| 54 | NEG | + | 12 | + | >160 | + | 32 | + | 24 | + |
| 58 | NEG | + | 12 | + | 10 | + | 32 | + | 40 | + |
| 66 | NEG | + | 24 | + | 10 | + | 36 | + | 20 | + |

Additional file 6C: Neutralising antibody titres to HYV and BEFV in sera collected from sentinel cattle recruited at Beatrice Hill Farm or Berrimah Farm in 2011-2012.

| **Location** | **Sentinel animal number** | **Serum collection date** | **HYV neutralising antibody titre** | **BEF neutralising antibody titre** |
| --- | --- | --- | --- | --- |
| Beatrice Hill Farm | 6 | 27 Aug 2012 | Negative | Negative |
|  |  | 8 Oct 2012 | Negative | Negative |
|  | 21 | 26 Sep 2012 | Negative | Negative |
|  |  | 3 Oct 2012 | 10 | ≥10* |
| Berrimah Farm | B01 | 23 Apr 2012 | Negative | Negative |
|  |  | 10 May 2012 | >160 | ≥10* |
|  | B03 | 10 May 2012 | Negative | Negative |
|  |  | 3 Dec 2012 | 80 | Negative |
|  |  | 4 Jan 2013 | 18 | Negative |
|  | B12 | 6 May 2011 | Negative | Negative |
|  |  | 6 Jun 2011 | Negative | Negative |
|  |  | 5 Dec 2011 | Negative | Negative |
|  |  | 24 Jan 2012 | 18 | Negative |
|  |  | 25 May 2012 | >160 | >160 |
|  | B16 | 19 Jan 2012 | Negative | 160 |
|  | B18 | 12 Sep 2011 | Negative | Negative |
|  |  | 1 Feb 2012 | 96 | Negative |
|  |  | 16 May 2012 | >160 | >160 |
|  | B40 | 12 Nov 2012 | Negative | Negative |
|  |  | 23 Nov 2012 | Negative | Negative |
|  | B44 | 16 Nov 2012 | 128 | ≥10* |

*+ denotes samples that were screened and found positive for BEFV neutralising antibodies at 1:10 only.
